# Exosome-mediated repair of spinal cord injury: cellular sources, mechanisms of action, and combined therapeutic strategies

**DOI:** 10.3389/fneur.2025.1645457

**Published:** 2025-10-13

**Authors:** Zaihong Cha, Yu Li, Jianeng Pu, Yuansheng Zhang, Qixiong Lu, Wei Huang, Tao Li, Xiaoyang Lu

**Affiliations:** ^1^The Affiliated Hospital of Kunming University of Science and Technology, The First People's Hospital of Yunnan Province, Kunming, Yunnan, China; ^2^Department of Neurosurgery, The First People's Hospital of Yunnan Province, The Affiliated Hospital of Kunming University of Science and Technology, Kunming, Yunnan, China; ^3^Research Center for Clinical Medicine, The First People's Hospital of Yunnan Province, The Affiliated Hospital of Kunming University of Science and Technology, Kunming, Yunnan, China; ^4^Institute of Neurosurgery and Neuroscience, The First People's Hospital of Yunnan Province, The Affiliated Hospital of Kunming University of Science and Technology, Kunming, Yunnan, China; ^5^Yunnan Province Spinal Cord Disease Clinical Medical Center, The First People's Hospital of Yunnan Province, The Affiliated Hospital of Kunming University of Science and Technology, Kunming, Yunnan, China

**Keywords:** spinal cord injury, exosomes, nerve repair, immunoregulation, miRNA, NDDS

## Abstract

Spinal cord injury (SCI) presents a significant clinical challenge due to its complex pathology and limited capacity for self-repair, often resulting in substantial physical dysfunction. Conventional treatments emphasize symptom management, yet usually fail to achieve nerve regeneration and full functional recovery. Recently, Exosomes(Exos) have gained attention as key modulators in biological processes such as immune regulation, intercellular communication, and tissue repair, showing promise in nerve injury and regeneration. This review synthesizes recent research on Exosome-based SCI therapies, including their biological origins, mechanisms, potential applications, and current limitations. Although Exos' research in SCI is nascent, early studies indicate promising safety and efficacy. Future studies are encouraged to delve deeper into Exos preparation, optimization, and delivery to maximize therapeutic effectiveness, potentially advancing SCI treatment options.

## 1 Introduction

SCI is a spinal cord dysfunction resulting from external trauma or disease, which frequently leads to sensory and motor dysfunction below the injury level (1–3). It also significantly impacts the normal functioning of the autonomic nervous system, causing patients to face numerous challenges such as paralysis, sensory loss, and disruptions in basic life activities like breathing and heartbeat (4). Consequently, this greatly diminishes the quality of life for patients while imposing a substantial burden on their families and society (5, 6). The incidence rate of traumatic spinal cord injury (TSCI) is estimated at 26.48 per 1 million people, whereas non-traumatic spinal cord injury (NTSCI) occurs at a rate of 17.93 per 1 million people. Notably, Central and Eastern Europe as well as Central Asia exhibit significantly higher rates of SCI compared to other regions worldwide; moreover, male patients constitute a much larger proportion than female patients (7, 8). When SCI occurs, it damages the blood-spinal cord barrier (BSCB), leading to complex pathophysiological changes including local metabolic disorders, calcium overload, inflammation, oxidative stress, iron death, apoptosis, glial scarring, neuroplasticity changes, and autonomic nervous dysfunction (1, 9). These interconnected chain reactions exacerbate patient conditions and rehabilitation difficulties. Currently, within the medical field, active efforts are being made to explore effective treatments for spinal cord injuries. However, despite existing treatment methods such as surgical decompression, drug therapy, and postoperative rehabilitation training being able to alleviate symptoms to some extent, they often yield unsatisfactory results in terms of neurological function recovery (6, 10). Therefore, finding new, more effective treatment approaches has become an urgent issue that needs addressing within the medical field.

With the continuous advancement of scientific research, Exos have emerged as a promising therapeutic strategy for SCI treatment (11–13). Exos are small vesicles secreted by cells that contain a diverse array of bioactive substances such as lipids, proteins, nucleic acids, and cytokines (14). They play crucial roles in intercellular communication and regulation while influencing the microenvironment of the injured spinal cord (15, 16). Numerous studies have demonstrated that Exos derived from various cell types, including mesenchymal stem cells, neural stem cells, and macrophages, can effectively facilitate nerve repair following SCI (17, 18). These Exos exert their effects through multiple mechanisms, including the inhibition of ferroptosis and apoptosis, as well as the promotion of axon regeneration. Moreover, they also exhibit anti-inflammatory properties while regulating glial scar formation and providing nutritional support to damaged nerve cells (16, 19), thereby instilling renewed hope for SCI treatment. Notably, microRNAs (miRNAs) contained within Exos have exhibited significant potential in the therapeutic management of SCI (20). Furthermore, due to its non-toxic nature upon infusion along with easy accessibility and absence of ethical concerns (18), Exos has emerged as an alternative to cell-based therapies offering improved safety profiles and enhanced therapeutic efficacy across various regenerative applications (21, 22). Concurrently, hypoxic preconditioning has been shown to enhance the secretion of Exos, thereby further augmenting their therapeutic efficacy (23, 24). Exos derived from hypoxic preconditioning exhibit particularly enhanced therapeutic potential. Lastly, as natural nanocarriers, Exos possess intrinsic advantages, including stable physical and chemical properties, low immunogenicity, and superior penetration capabilities across the blood-brain barrier (BBB) and BSCB. These characteristics render them an ideal candidate for nanotherapeutic applications (25, 26). The utilization of Exos as carriers in the construction of nanomedical drug delivery systems (NDDS), combined with biological scaffolds in a synergistic therapy approach (27–29), has not only improved the therapeutic effectiveness of Exos in SCI treatment but also overcome the limitations associated with single therapy.

Although the research on Exos treatment for SCI is still in a continuous development phase, the existing research findings have established a solid foundation for future clinical application (30, 31). Further investigation into the mechanisms underlying Exos treatment for SCI, alongside the optimization of Exos preparation and delivery methods (32, 33), as well as the assessment of its long-term efficacy and safety (21, 34), are critical steps necessary to facilitate the translation of this therapy into clinical practice. At present, the therapeutic strategy based on Exos has shifted from single-molecule regulation to multimodal collaborative intervention, such as local sustained release in combination with light-curing hydrogels, or the construction of engineered Exos with enhanced functions through gene editing technology (35, 36). It is essential to conduct additional high-quality studies to advance the clinical application of Exos treatment for SCI, thereby offering new hope and effective therapeutic strategies for patients with SCI.

## 2 Exosomes

**Figure 1:**
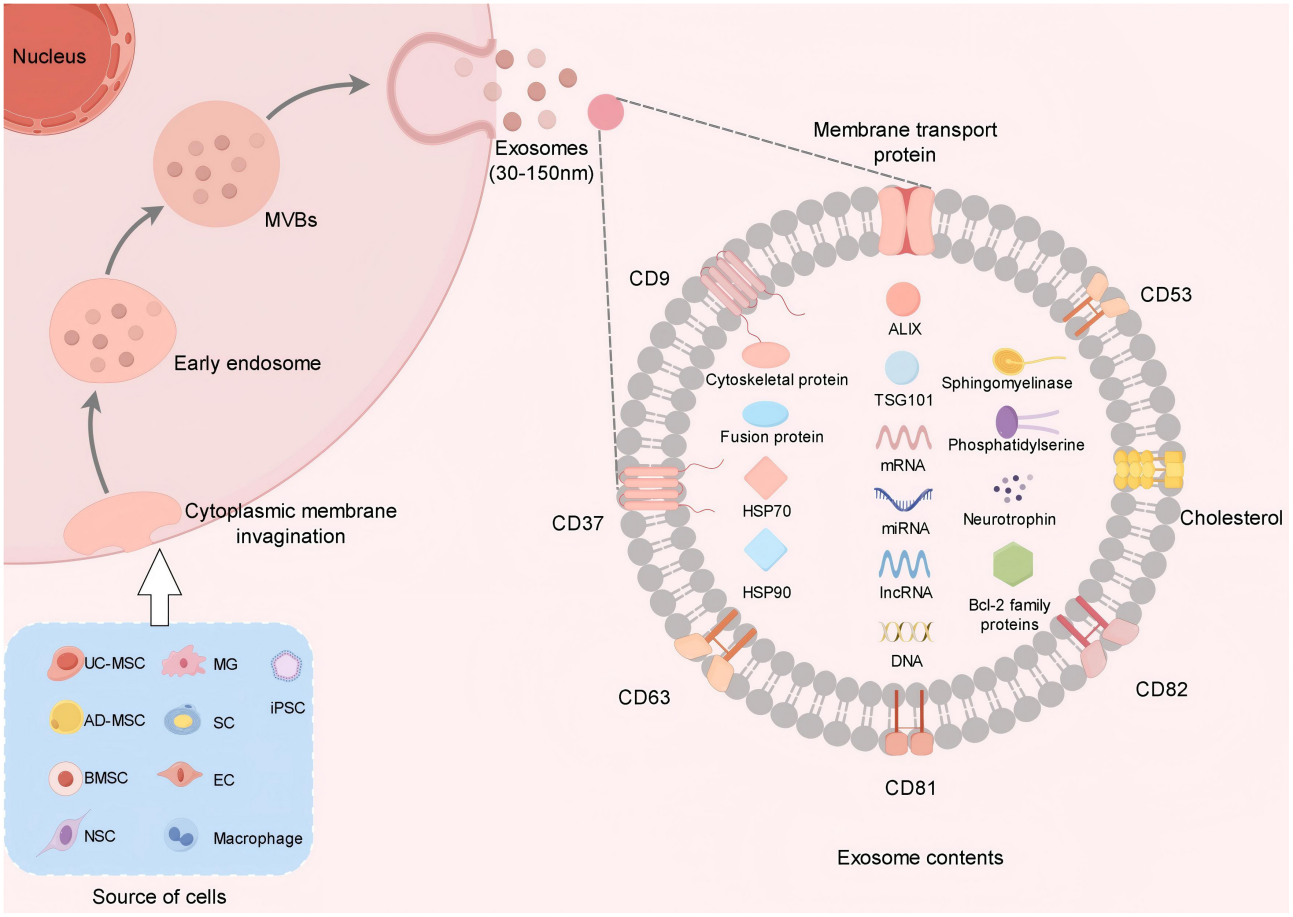
Exos are derived from a variety of cells. Exos are released after the fusion of MVBs with the cell membrane. Its contents include protein, RNA and lipids. Proteins are involved in signal transduction, RNA regulates gene expression, and lipids provide structural support.

Exos are nanoscale membrane vesicles secreted by cells (37), typically measuring between 30 and 150 nm in diameter (33). The formation of Exos begins in the endosomal system within the cell, where the cytoplasmic membrane invagination forms the early endosomes, and the early endosomes further mature to form the late endosomes, also known as multivesicular bodies (MVBs). MVBs fuse with the plasma membrane of the cell and release the small vesicles contained within them into the extracellular environment, which are called Exos (38–40). Exos encompass a diverse array of biomolecules, including proteins (such as cytoskeletal proteins, membrane transport and fusion proteins, and members of the tetraspanin family like CD9, CD37, CD53, CD63, CD81, and CD82), lipids (including cholesterol, sphingomyelin, and phosphatidylserine), and nucleic acids (such as DNA, mRNA, miRNA, lncRNA, and circRNA) (15, 41, 42). Additionally, they contain proteins such as ALIX, TSG101, and heat shock proteins (HSP70, HSP90), which serve as markers and are involved in Exosome biogenesis (39, 43, 44) (Figure 1). The composition of Exos reflects the physiological and pathological states of their originating cells and can be transferred to recipient cells to facilitate various biological functions. Based on the extent of artificial modification, Exos can be broadly categorized into natural Exos and engineered Exos (28). A diverse array of human cells, such as stem cells, Schwann cells (SC), endothelial cells (EC), macrophages, microglial cells (MG), and even tumor cells (18), are capable of producing Exos. Exos from these various cell types may offer unique therapeutic benefits in treating SCI. In recent years, induced pluripotent stem cell (iPSC) -derived Exos have become a research hotspot due to their unlimited proliferation, multi-directional differentiation, and personalized treatment potential. It has been confirmed that iPSC-Exos can effectively promote the polarization of M1 macrophages to anti-inflammatory M2 macrophages by targeting hepatocyte growth factor (HGF) by delivery of miR-199b-5p, and enhance nerve regeneration through the PI3K signaling pathway (45). Another group further developed a gene-edited engineered exosome that could be targeted to the site of SCI by intranasal delivery of BDNF-overexpressing mesenchymal stem cell exosomes (BDNF-sEV) to significantly promote neurological recovery in rat and monkey models. In addition, hypoxic preconditioning was confirmed to significantly enhance the therapeutic effect of MSC-derived Exos, and the mechanism was related to enhancing the activities of antioxidant enzymes and promoting the secretion of angiogenic factors (46). Liang et al. (47) found that hypoxic preconditioning of bone marrow mesenchymal stem cell-derived exosomes (BMSC-HSEV) inhibited the IRAK1/TRAF6/NF-κB pathway by carrying miR-146a-5p, which could effectively regulate macrophage polarization and alleviate SCI.

In summary, the diversity of Exos sources plays a critical role in determining their biological functions and potential clinical applications, underscoring their importance as a key medium for intercellular communication. Each Exos source offers unique advantages and limitations within specific research and application contexts. Therefore, selecting the appropriate Exos source is essential for optimizing their utilization in medical and biological fields (Table 1).

**Table 1:** Comparison of advantages and disadvantages of Exos from different cell sources.

**Different cell sources**	**Main functions/research directions**	**Source and acquisition difficulty**	***In vitro* preparation and amplification capabilities**	**Pathologic and immunogenicity**	**Primary competitive advantages**	**Main limitations**	**Clinical translational potential**
umbilical cord mesenchymal stem cell (UC-MS) (111, 155–157)	Tissue repair Immune regulation, and inflammation suppression	Umbilical cord Huatong's gel, easy to obtain (postpartum waste, non-invasive)	It is easy to separate, has strong amplification ability and can be cultivated on a large scale	Low immunogenicity (Low expression of HLA-DR)	Rich in sources and with little ethical controversy; Strong paracrine function; High security	The functional activity is slightly lower than that of other MSCS. The proliferation ability decreases after long-term passage	Relatively high (multiple clinical trial stages)
adipose-derived mesenchymal stem cell (AD-MS) (157–160)	Soft tissue repair, metabolic regulation, and immune regulation	Adipose tissue is relatively easy to obtain (liposuction, minimally invasive)	The volume of adipose tissue is large and the efficiency of separation and amplification is high	Low immunogenicity	High yield (a large number of cells can be obtained from each gram of fat); Rich in paracrine factors	The function is affected by the donor's age and obesity status. It is prone to aging outside the body	Relatively high (in some clinical applications)
bone marrow mesenchymal stem cell (BMSC) (72, 157, 161, 162)	Bone/cartilage repair, hematopoietic support, and nerve regeneration	Bone marrow, moderate acquisition (puncture sampling, invasive)	The separation difficulty is moderate, and the amplification capacity is moderate	Low immunogenicity	The most in-depth research and clear functions; It has strong osteogenic/chondrogenic differentiation ability	The materials are creative. Low content in bone marrow	Relatively high (with relatively mature clinical application)
neural stem cell (NSC) (163–165)	Neural regeneration, synaptic repair, and neural circuit reconstruction	Embryonic brain tissue/adult brain regions are difficult to obtain (with significant ethical controversy)	The separation and purification are complex, and amplification is limited (prone to differentiation)	Moderate immunogenicity (Allogeneic transplantation may cause rejection)	It has strong targeting ability and can directly differentiate into neurons/glial cells	The risk of tumor formation is relatively high; Adult-derived cells have weak proliferation ability	Moderate (mostly in basic research)
microglial cell (MG) (166–168)	Neuroimmune regulation, Aβ clearance, and injury repair	Brain tissue is difficult to obtain (primary isolation is difficult)	*In vitro* culture is easy to activate (phenotypic unstable)	Moderate immunogenicity (Enhanced immune activity under pathological conditions)	Precise regulation of neuroinflammation; Participate in the pathological process of neurodegenerative diseases	Dual functionality (it can both protect and cause damage); Difficult to maintain externally	Moderate (mechanism research stage)
schwann cell (SC) (169–172)	Peripheral nerve regeneration, axon guidance, and myelin formation	Peripheral nerves (such as the sciatic nerve) are more difficult to obtain (surgical sampling is required)	The separation and purification steps are complex and the amplification capacity is limited	Low immunogenicity	The “gold standard” cells for peripheral nerve repair; It has a strong ability to promote axonal regeneration	Limited source; Its effect on the repair of the central nervous system is unknown	Relatively high (Clinical application of peripheral nerve repair)
endothelial cell (EC) (71, 101, 173)	Angiogenesis, improvement of local blood supply, and repair of the BBB	Vascular tissue/pluripotent stem cell induction, moderate acquisition	Specific selection of culture medium is required, and the purification is difficult	Moderate immunogenicity (expressing vascular endothelial antigen)	Promote revascularization of ischemic tissue; Maintain vascular homeostasis	The effect is limited when applied alone. It is prone to form abnormal vascular networks	Moderate (often used in combination with other cells
Macrophagocyte (94, 174, 175)	Immune regulation, inflammation clearance, and tissue remodeling	Peripheral blood mononuclear cell induction, moderate acquisition	Monocytes are easy to isolate and have poor controllability in inducing differentiation	Moderate immunogenicity (affected by polarization state)	Strong ability to remove necrotic tissue; The M1/M2 phenotypic transition can be regulated	Strong functional plasticity (easily disturbed by the micro-environment); Risk of inflammation	Moderate (Immune-related disease research)
induced pluripotent stem cells (iPSCs) (176–178)	Personalized treatment, multi-directional differentiation (such as nerve cells, myocardial cells)	Adult cells (such as skin fibroblasts) are relatively easy to obtain	Reprogramming technology is complex and the differentiation steps are cumbersome	Low immunogenicity (Self-origin can avoid rejection)	It can differentiate into any cell type; Suitable for personalized medicine	Tumorigenic risk (genomic instability); The preparation cycle is long (4 to 6 weeks)	High (Great potential, many challenges)

## 3 Mechanism of exosomes in the treatment of SCI

The potential of Exos in the treatment of SCI is evident, and the subsequent mechanisms of action are as follows (Table 2):

**Table 2:** Summary of the mechanisms of exosomes therapy for spinal cord injury.

**Mechanism of action**	**Core function**	**Key molecules/components**	**Involving signal pathways**
Promote neuroprotection	1. Inhibition of neuronal apoptosis: blocking the mitochondrial apoptotic cascade by activating survival signaling pathways and transmitting anti-apoptotic proteins; 2. Enhanced neuronal survival: enhanced neuronal tolerance to injury through activation of intracellular protective pathways by neurotrophic factors; 3. Inhibition of ferroptosis: reduce iron accumulation in neurons, enhance antioxidant capacity, and block lipid peroxidation; 4. Promote functional recovery: targeted delivery of high concentration of nutritional factors to improve the efficiency of nerve repair at the injured site.	1. Neurotrophic factors: BDNF, NGF, GDNF 2. Anti-apoptotic proteins: Bcl-2, Bcl-xL Heat shock proteins: HSP70, HSP90 3. miRNAs: miR-21a-3p, miR-27a-3p; 4. Iron death regulatory molecules: PINK1, Parkin (related to mitochondrial phagocytosis), Nrf2 (antioxidant transcription factor), GCH1 (4-hydroxypterin synthase), BH4 (4-hydroxypterin); 5. Others: MaR1 (anti-inflammatory and regenerative) Nrg1 (myelin protection), Natural products (resveratrol, 7,8 - dihydroxyflavone, propofol, chuanxiong chenpiine, etc.).	Survival and anti-apoptosis pathways: MAPK (ERK/JNK/p38); PI3K/Akt/mTOR TLR4/MyD88/NF-κβ 2. Ferroptosis regulatory pathway: PINK1/Parkin/mitochondrial phagocytosis Nrf2/ARE/GCH1/BH4 3. Neurotrophic factor pathway: BDNF/TrkB/MAPK/PI3K/Akt
2. Promote axon regeneration and synaptic remodeling	1. Axonal growth and extension: enhanced axonal extension by promoting microtubule/neurofilament assembly through axon growth factors; 2. Myelin regeneration and protection: regulating oligodendrocyte differentiation, promoting myelin formation, and protecting axon structure; 3. Synapse formation and circuit reconstruction: promote the expression of synapse-associated proteins, regulate neural connections, and restore signal transduction; 4. Relieve the inhibition of regeneration: regulate glial scar-related molecules to reduce axonal growth retardation.	1. Axonal growth factors: Neurofilament proteins (such as NF-L, NF-M), Microtubule-associated proteins (such as MAP2, Tau); 2. miRNA: miR-26a, miR-199-5p, miR-431-3p; 3. Ubiquitin ligases and associated molecules: Neural precursor cell expressed developmentally downregulated protein 4 (NEDD4), NEDD4-1, NEDD4-2, Ndfip1, Ndfip2 (NEDD4-binding proteins), Roundabout (Robo) receptor (axonal guidance receptor); 4. Others: Exercise training synergy factor (enhanced through the JNK1/c-Jun pathway).	1. Axonal regeneration pathway: miR-199-5p / Inhibiting PTEN/PI3K/Akt/mTOR; NF-κβ / Promoting M2 polarization of microglia / Enhancing the recruitment of NSC; 2. Synaptic remodeling pathway: JNK1/c-Jun / Regulating the expression of synaptic-related genes (such as synaptophysin, PSD95) 3. Axonal guidance pathway: NEDD4/Robo receptor ubiquitination / Proteasome degradation / Removing axonal growth inhibition
3. Inflammation suppression and immune regulation	1. Inhibit the release of pro-inflammatory factors: Reduce inflammatory factors such as TNF-α, IL-1β, and IL-6, and alleviate nerve cell damage; 2. Regulate macrophage polarization: Promote M2-type (anti-inflammatory and repair-type) polarization and inhibit M1-type (pro-inflammatory and damaging) activation; 3. Enhance anti-inflammatory cell function: Through Treg cell-derived Exos to inhibit excessive immune response; 4. Synergize with drug effects: combine with ibrutinib to block excessive neural-immune activation.	1. miRNA: miR-23a-3p, miR-222-3p, miR-216a-5p, miR-2861, miR-709; 2. Immune regulatory molecules: SOCS3, JAK2, STAT3, IRAK1; 3. Immune cell-related: M2 type macrophage markers (CD206, IL-10), Treg cells; 4. Others: Bruton's tyrosine kinase (BTK), ibrutinib (BTK inhibitor).	1. Macrophage polarization pathway: SOCS3 / Inhibiting JAK2/STAT3; ROS/MAPK/NF-κβ P65; 2. Neuroinflammation regulation pathway: miR-2861 / Inhibiting IRAK1/TLR4/NF-κβ; BTK / Inhibiting microglia/astrocyte activation (Ibrutinib combined mechanism)
4. Promote angiogenesis and maintain BSCB integrity	1. Angiogenesis: Promotes the proliferation, migration, and lumen formation of vascular endothelial cells at the injured site, improving blood supply; 2. BSCB repair: Enhances the stability of tight junctions, reduces vascular permeability, and prevents inflammatory cells / harmful substances from invading; 3. Microcirculation improvement: Mediated by NO, it causes vasodilation and enhances the oxygen and nutrient supply in the injured area.	1. Angiogenic factors: VEGF, FGF, PDGF; 2. miRNA: miR-210, miR-501-5p; 3. Proteins: OTULIN (deubiquitinating enzyme, activates Wnt pathway), tight junction proteins (Claudin-5, Occludin, ZO-1), Janus kinase 1 (JAK1), signal transducer and activator of transcription 3 (STAT3), myosin light chain kinase (MLCK); 4. Others: Hypoxia-inducible factor - 1α (HIF-1α, related to hypoxia preconditioning).	Angiogenesis pathway: PI3K/Akt/eNOS; OTULIN / Activates Wnt/β-catenin/VEGF; HIF-1α / VEGF; 2. BSCB repair pathway: miR-210 / JAK1 / STAT3 / Expression of tight junction proteins; miR-501-5p / Inhibits MLCK / Reduces degradation of tight junction proteins
5. Regulation of the extracellular matrix	1. ECM remodeling: Regulates the synthesis and degradation of components such as collagen and fibronectin, maintaining the stability of the matrix structure; 2. Gliosis inhibition: Reduces the deposition of chondroitin sulfate proteoglycans (CSPG), inhibits the activation of type A astrocytes; 3. Microenvironment improvement: Regulates matrix metabolism through the balance of MMPs/TIMP, creating a favorable environment for neural regeneration; 4. Barrier protection: Regulates proteins related to the blood-brain-spinal cord barrier, reducing ECM damage.	1. Matrix regulatory molecules: Matrix metalloproteinases (MMPs, such as MMP-2, MMP-9), tissue inhibitor of metalloproteinases (TIMP, such as TIMP-1, TIMP-2), ADAMTS (polypeptide proteases that degrade CSPG); 2. miRNA: miR-467b-3p (carried by UTX–/–EC-Exos); 3. Scar-related molecules: Chondroitin sulfate proteoglycan (CSPG), A1 type astrocyte marker (complement C3), Rab27a (small G protein that mediates CSPG release); 4. Others: Transforming growth factor β (TGF-β, promotes vascular stability), arginine-glycine-aspartic acid (RGD, targeting-modifying peptide), phosphatase and tensin homolog (PTEN, inhibited by miR-467b-3p)	1. ECM remodeling pathway: MMPs/TIMP balance / Regulating collagen / Fibronectin degradation and synthesis; miR-467b-3p / Inhibiting PTEN/PI3K/Akt/mTOR (Promoting M2 macrophages / Reducing ECM destruction) 2. Scar inhibition pathway: Inhibiting A1- type astrocyte activation / Reducing CSPG synthesis; ADAMTS / Degradation of CSPG / Inhibiting RhoA/ROCK (Relieving axonal inhibition)

### 3.1 Promote neural protection

**Figure 2:**
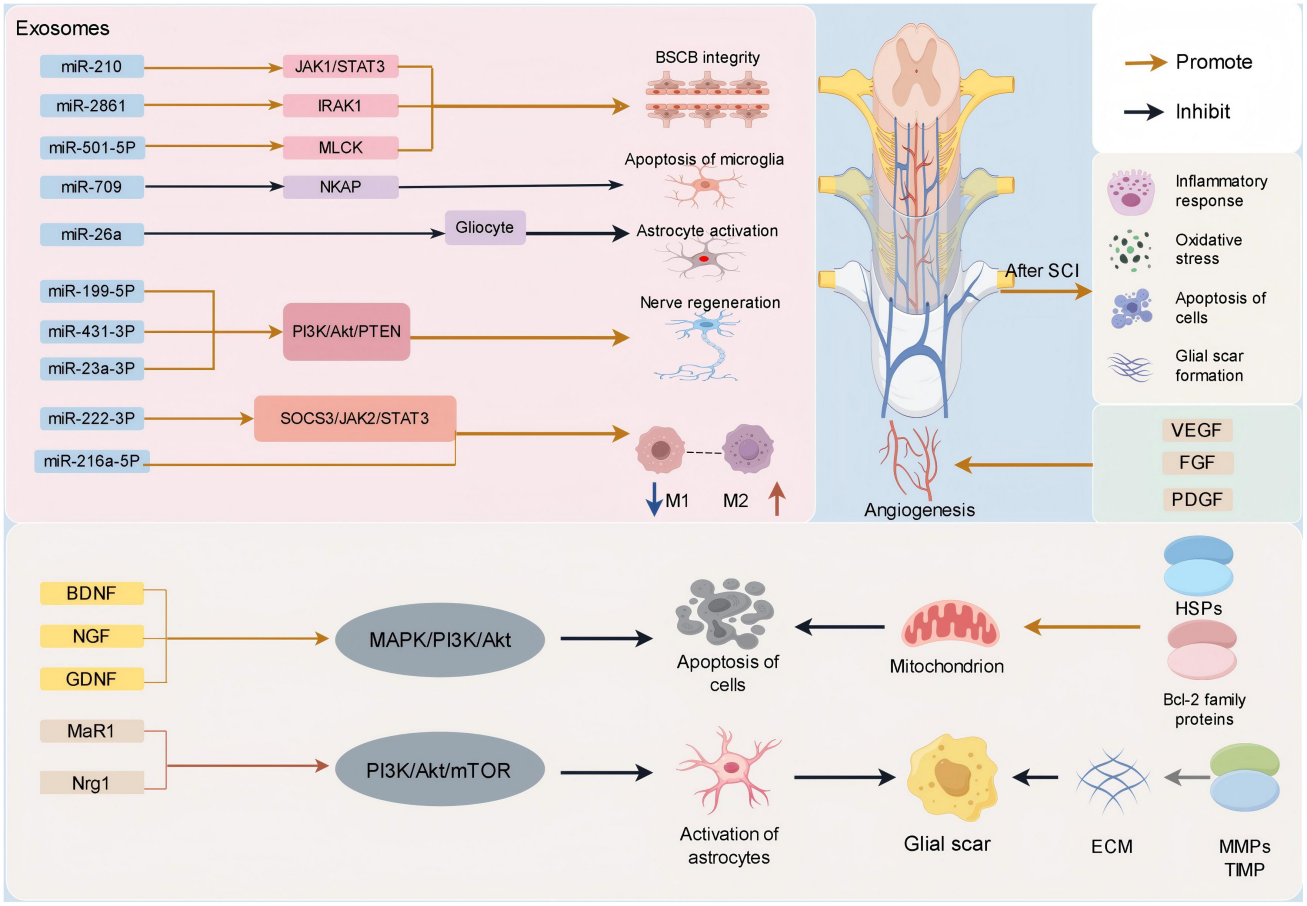
SCI can trigger a series of complex cascade reactions, including inflammatory response, oxidative stress, apoptosis, glial scar formation, etc. Exos can treat SCI through a variety of mechanisms. It can carry biological active molecules such as miRNA, neurotrophins, cytokines, Bcl-2 family proteins, HSPs anti-apoptotic proteins, and so on. It plays an important role in inhibiting inflammatory response, inhibiting glial scar formation, promoting nerve cell survival and axon regeneration, inhibiting cell apoptosis, promoting angiogenesis, and maintaining the integrity of BSCB.

Exos encompass a diverse array of neurotrophic factors, including brain-derived neurotrophic factor (BDNF), nerve growth factor (NGF), and glial cell-derived neurotrophic factor (GDNF) (48, 49) (Figure 2). These neurotrophic elements are capable of activating intracellular survival signaling pathways, such as the mitogen-activated protein kinase (MAPK) and phosphatidylinositol 3-kinase (PI3K)/protein kinase B (Akt) pathways. Such activation inhibits the initiation of apoptotic processes and enhances the survival capacity of neuronal cells (50). In addition, Exos contain anti-apoptotic proteins, including members of the B-cell lymphoma-2 (Bcl-2) family and heat shock proteins (HSPs) (Figure 2). These proteins modulate mitochondrial function, stabilize mitochondrial membrane potential, and inhibit the release of cytochrome C, thereby obstructing the apoptotic cascade and reducing neuronal apoptosis (51–53).

Fu et al. (54) demonstrated that Exos derived from human adipose tissue mesenchymal stem cells (MSCs) inhibit neuronal apoptosis and promote neurogenesis via the miR-21a-3p/PI3K/Akt signaling pathway. Zhu et al. developed a nanofiber scaffold composed of a hyaluronic acid hydrogel patch designed to deliver Exos and methylprednisolone to the injured spinal cord in a non-invasive manner. This approach effectively inhibited inflammation and neuronal apoptosis while enhancing neuronal survival through the modulation of TLR4/MyD88/NF-κB, MAPK, and Akt/mTOR pathways (55). Furthermore, Maresin 1 (MaR1), recognized as an anti-inflammatory and pro-resolving mediator, exhibits potential for tissue regeneration. Wei et al. (56) found that MaR1 suppresses astrocyte activation via the PI3K/Akt/mTOR signaling pathway, reduces the production of pro-inflammatory cytokines in the spinal dorsal horn of mice, and facilitates the regeneration of injured nerves. Neuregulin-1 (Nrg1) is crucial for the differentiation of oligodendrocytes. Ding et al. demonstrated in a study involving SCI in rats that intravaginal administration of Nrg1 can induce the transformation of reactive astrocytes into oligodendrocyte lineage cells, a process mediated by the PI3K/Akt/mTOR signaling pathway. This pathway inhibits astrocyte proliferation, promotes myelin regeneration, and protects axons (57). Furthermore, natural compounds such as resveratrol, 7,8-dihydroxyflavones, propofol, and cephalin have been shown to exert neuroprotective effects through modulation of the PI3K/Akt/mTOR signaling pathway (58). The potential for combining Exos with these substances to treat SCI and achieve a synergistic therapeutic effect presents a promising strategy for the regulation of nerve damage, warranting further investigation.

In recent years, a breakthrough has been made in the mechanism research of Exos in the field of neuroprotection. Pay, etc. confirmed that the high expression of BDNF nasal delivery non-greeks secrete body (MSCs–sEV) can be targeted enrichment in SCI, its concentration of neurotrophic factor is a natural body secretion of eight times, in the rhesus monkey model, to realize motor function recovery rate was 67% (46). Sun et al. also found that nasal delivery of a specific subset of MSC-derived small extracellular vesicles, CD146+CD271+ ucmsc-sev, could target and enrich at the site of SCI and inhibit DLL4 through the transfer of miR-27a-3p to regulate inflammation, inhibit apoptosis, and promote nerve regeneration. It can effectively reduce traumatic SCI and improve neurological function recovery (59). In addition, Exos have shown remarkable potential for inhibiting iron death and thus promoting neuroprotection (60, 61). Exos can play a role by transferring specific proteins, miRNAs, and other molecules, regulating iron metabolism, enhancing antioxidant capacity, and regulating related signaling pathways. These mechanisms, on the one hand, reduce the accumulation of iron ions in neurons, on the other hand, alleviate oxidative stress and lipid peroxidation, and ultimately inhibit ferroptosis (62, 63). Zhang et al. (64) were the first to show that *in vitro* Exos therapy activates mitochondrial phagocytosis via the PINK1/Parkin pathway, thereby reducing ferroptosis in neuronal cells, which plays a crucial role in neuroprotection following trauma. Similarly, Chen et al. (65) demonstrated that mesenchymal stem cell-derived Exosomes (MSC-Exos) alleviate ferroptosis in microglia through the Nrf2/GCH1/BH4 signaling pathway, indicating their promising potential in protecting and restoring neural function after SCI.

Consequently, Exos exhibit anti-apoptotic and neuroprotective properties through the transmission of anti-apoptotic signaling molecules, modulation of intracellular signaling pathways, and inhibition of ferroptosis. These mechanisms suggest a wide range of potential applications for Exos in the treatment of neurological disorders.

### 3.2 Promote axon regeneration and synaptic plasticity

Exos play a crucial role in axon regeneration and synaptic remodeling (66). They are enriched with various axon growth factors, including neurofilament and microtubule-associated proteins (MAPs) (Figure 2), which are instrumental in facilitating axonal growth and extension (67, 68). Concurrently, the transfer of RNA is critical in tissue formation. Exos are rich in diverse miRNAs that can modulate gene expression to enhance axon regeneration and myelination (69) (Figure 2). In a study by Gao et al. (70) the delivery of miR-26a to damaged neurons via an *in vivo* regenerative system led to decreased astrocyte activation at the injury site and promoted neuronal axon growth. Furthermore, research by Huang et al. demonstrated that Exos derived from endothelial cell (EC) culture medium can activate the PI3K/Akt/PTEN signaling pathway by upregulating miR-199-5p, thereby facilitating nerve regeneration. Their findings also indicated that EC-derived Exos exhibit strong neuronal affinity both *in vitro* and *in vivo* (71). In a study utilizing the SCI model, Sun et al. successfully isolated Exos characterized by CD271+CD56+ markers from a specific CD271+CD56+ bone marrow stromal cell (BMSC) subgroup through on-site implantation. Their findings indicated that miR-431-3p plays a crucial role in the mechanism by which CD271+CD56+ BMSC-derived Exos facilitate functional recovery and axonal regeneration post-SCI (72). Similarly, Fan et al. identified that BMSC-derived Exos modulate M2 polarization of microglia via the NF-κB signaling pathway, resulting in a marked decrease in CD68-positive microglia, enhanced recruitment of local neural stem cells (NSCs), and increased axonal growth through the PTEN/PI3K/Akt/mTOR pathway. This process significantly contributes to early functional recovery in mouse models of SCI (73). Additionally, other research has demonstrated that exercise training may work synergistically with BMSC-derived Exos to regulate neuronal apoptosis via the JNK1/c-Jun signaling pathway, thereby reconstructing neural circuits, promoting synaptic formation and axonal regeneration, and ultimately enhancing neural function recovery (74).

In recent years, breakthroughs have been made in the molecular mechanism of Exos in synaptic remodeling, which has become the core strategy for SCI repair by regulating the dynamic balance of synaptic structure and function at multiple levels. Postsynaptic density (PSD) is a key structure in synaptic plasticity, and its dynamic assembly depends on protein phase separation. The study by Zhang's team found that the scaffold protein SAPAP carried by mesenchymal stem cell Exos regulates the fusion and separation of PSD core and PSD pallium through phosphorylation: when the phosphorylation level of SAPAP increased, PSD core fused with PSD pallium to form a homogeneous concentrated phase, which enhanced the aggregation of NMDA receptors on the postsynaptic membrane. Under low phosphorylation, the PSD structure dissociates to maintain synaptic stability. In the SCI rat model, exosome intervention increased the PSD volume by 2.1 times and the synaptic transmission efficiency by 40% (75). Another study confirmed that the postsynaptic density protein 95 (PSD-95) carried by Exos of neural stem cells could participate in the assembly of PSD and promote the recovery of synaptic connection strength in the injured area (76). In addition, RVG-BDNF-Exos (BDNF-targeted delivery Exos modified by rabies virus glycoprotein) developed by Cheng's team penetrated the BBB after tail vein injection, specifically bound to nicotinic acetylcholine receptors on the surface of neurons, and delivered the BDNF gene to postsynaptic neurons. The Exos significantly up-regulated the expression of PSD95 and Syn-1 in the hippocampus and the injured area of the spinal cord, restored the synaptic density to 68% of the normal level, and reversed the synaptic loss by activating the TrkB/ERK pathway. A similar strategy improved axonal regeneration to 58% in a macaque model of SCI, demonstrating the clinical potential of targeted delivery for the first time in a non-human primate (77). According to Piette et al. (78) neural energy metabolism is closely related to synaptic plasticity. Kochan et al. found in animal experiments that there will be a transient surge in mitochondrial fusion dynamics when newborn neurons enter the critical period. This process can stabilize the elongated mitochondrial morphology in dendrites and provide energy support for synaptic plasticity, which is crucial for the plasticity of new synapses and the improvement of existing brain circuits (79).

In addition, neural precursor cells expressing developmentally down-regulated protein 4 (NEDD4) combined with Exos may play an important role in the treatment of SCI. After wrapping NEDD4 in Exos, NEDD4 can be transported to related cells at the site of injury, such as neurons and glial cells. Thus, it can play a more effective role in relieving the inhibition of nerve regeneration and regulating the microenvironment of nerve regeneration (80, 81). NEDD4, as an E3 ubiquitin ligase, is involved in the regulation of axon guidance during neural development. NEDD4-1 and NEDD4-2 were found to be required for axon guidance at the spinal commissural, and they regulate Roundabout (Robo) receptor endocytosis, ubiquitination, and degradation by interacting with Ndfip1 and Ndfip2 proteins to form a complex. Robo receptor is an axon guidance receptor that plays an important role in axon growth cone guidance (82, 83). Ding et al. (84) discovered that Nedd4 is required for developmental myelination by stabilizing the E3 ligase VHL through K63-linked ubiquitination, revealing a new role for Nedd4 in glial biology. Fimiani et al. (85) demonstrated in animal experiments that Nedd4 is required for the correct accumulation of differentiated oligodendrocytes and can promote myelination in the central and peripheral nervous systems of mice. Sullivan and Bashaw et al. (86) demonstrated that commissuless (Comm) promotes the growth of the axon midline by promoting Robo1 ubiquitination of Nedd4 and eventually leading to its degradation. Shi et al. (87) also found in animal experiments that NEDD4 may control the molecular mechanism of the endocytosis pathway and play an important role in the initiation stage of demyelination and axon regeneration. After SCI, NEDD4 may regulate related receptors through a similar mechanism, affect the regeneration and growth direction of axons, and promote the correct extension of axons at the injury site. In addition, it has been found that MiR-155-5p overexpression inhibits nuclear PTEN expression by targeting Nedd4 family interacting protein 1 (Ndfip1), which in turn aggravates astrocyte activation and glial scarring in SCI models (88). NEDD4 may regulate the expression and function of related proteins in glial cells, inhibit the over-expressed proteins that hinder nerve regeneration in the glial scar, and promote the secretion of some factors that are beneficial to nerve regeneration, thereby improving the microenvironment of nerve regeneration.

Exos have exhibited tremendous potential and diverse mechanisms in promoting nerve regeneration; however, there remain numerous unexplored areas that require further investigation to fully harness their role in nerve regeneration and disease treatment while advancing their development and application in clinical settings.

### 3.3 Inflammation suppression and immune regulation

The occurrence of inflammation is a prominent pathological process following SCI, and effective management of both local and systemic inflammation plays a pivotal role in enhancing patient prognosis (89, 90). SCI triggers a robust inflammatory response, leading to the release of numerous inflammatory mediators such as tumor necrosis factor-α (TNF-α), interleukin-1 (IL-1), and interleukin-6 (IL-6) (91, 92), thereby exacerbating neuronal damage (Figure 2).

In the event of SCI, macrophages accumulate at the injury site and play a pivotal role in the subsequent immune response (93). Macrophage-derived Exos significantly influence the immune microenvironment in SCI, with the miR-23a-3p/PTEN/PI3K/Akt signaling pathway potentially playing a critical role (94). Exos can modulate macrophage polarization by promoting the M2 subtype while inhibiting the activation of the M1 subtype. M2 macrophages exhibit anti-inflammatory properties and facilitate tissue repair, whereas M1 macrophages release pro-inflammatory cytokines that intensify inflammation (95–97). Ren et al. demonstrated that spinal cord-derived Exos can mitigate inflammation following SCI by suppressing M1 polarization and promoting M2 polarization. The SOCS3/STAT3 signaling pathway is essential in enhancing the inflammatory microenvironment and inhibiting neuronal apoptosis (98). Additionally, reactive oxygen species (ROS) can induce M1 macrophage polarization via the MAPK/NF-κB P65 signaling pathway. Liu et al. (99) reported that Exos derived from dental pulp stem cells ameliorate SCI by reducing M1 macrophage polarization through the ROS/MAPK/NF-κB P65 signaling pathway. Peng et al. (100) also observed that histone demethylase UTX deletion (UTX–/–EC) in endothelial cells promotes neural recovery mainly through Exos from UTX–/–EC polarizing macrophages toward an M2 subtype after SCI.

Moreover, bioactive molecules such as miRNAs and proteins within Exos may contribute to the modulation of inflammatory responses. Yuan et al. (101) reported that endothelial cell-derived Exosomes (EC-Exos) enhanced the prognosis of SCI via the SOCS3/JAK2/STAT3 signaling pathway, while the upregulation of miR-222-3p in EC-Exos led to a reduction in pro-inflammatory macrophages and an increase in anti-inflammatory macrophages. Liu et al. further demonstrated the potential involvement of miR-216a-5p in the polarization of microglial cells. Additionally, it has been observed that MSC-Exos produced under hypoxic conditions exert a more pronounced effect on neurological function recovery compared to normoxic MSC-Exos (24). Regulatory T (Treg) cells, recognized as potent anti-inflammatory agents, play a crucial role in mitigating neuroinflammation following SCI. Kong et al. (102) demonstrated that Exos derived from Treg cells can encapsulate and deliver miR-2861 to modulate IRAK1 expression, thereby influencing BSCB integrity and reducing neuroinflammation in murine models of SCI. Furthermore, Xiong et al. (103) confirmed through animal studies that Treg cells target NKAP with miR-709, leading to decreased microglial apoptosis and enhanced motor function recovery post-SCI. These findings suggest that by strategically designing and applying specific combinations of these miRNAs, synergistic effects could potentially enhance the effectiveness of SCI repair.

In addition, the activation of Bruton's tyrosine kinase (BTK) is associated with microglia/astrocytes and B-cell neuroimmune response mechanisms. Ibrutinib is a BTK inhibitor in innate immune cells. Torabi et al. (104) found that ibrutinib can reduce neutrophil infiltration, protect nerve tissue, and enhance the recovery of motor ability in SCI model mice. Yu et al. (105) also found in animal experiments that ibrutinib blocked excessive neuroimmune responses and promoted neuroprotection in SCI rat models through BTK-mediated activation of microglia/astrocytes and B cell/antibody responses. Therefore, Exos combined with ibrutinib may provide a new strategy for the treatment of SCI.

### 3.4 Promote angiogenesis and maintain BSCB integrity

Effective angiogenesis is essential for the reparative processes following SCI. Exos contain a variety of angiogenesis-related factors, including vascular endothelial growth factor (VEGF), fibroblast growth factor (FGF), and platelet-derived growth factor (PDGF) (106, 107). These factors facilitate the proliferation and migration of vascular endothelial cells and support the formation of vascular lumens at the site of injury, thereby ensuring an adequate supply of nutrients and oxygen necessary for the survival and regeneration of nerve cells (19, 108). Li et al. demonstrated that cerebrospinal fluid-derived Exosomes (CSF-Exos) can activate the PI3K/Akt signaling pathway, promoting vascular regeneration and enhancing motor function recovery post-SCI. This discovery indicates a potential novel therapeutic strategy for acute SCI (109). Additionally, Luo et al. (36) reported that Exos derived from M2 macrophages (M2-Exos) augment angiogenic activity *in vitro* by activating the Wnt/β-catenin signaling pathway through the transfer of OTULIN protein, thereby promoting vascular regeneration and functional recovery in murine models of SCI. Li et al. (23) on the other hand, effectively repaired neural tissue in mice by stimulating angiogenesis using Exos derived from human umbilical vein endothelial cells (HUVEC) through hypoxia pretreatment.

Furthermore, Exos exhibit the potential to facilitate the repair of the BSCB and preserve its integrity. In a study conducted by Gao et al. (110) the administration of Exos into mice with SCI demonstrated that miR-210 activates the JAK1/STAT3 signaling pathway, thereby modulating endothelial barrier function, enhancing BSCB integrity, and promoting the recovery of motor function. In another animal experiment conducted by Xie et al. (111) CD146+CD271+ MSC-Exos were found to upregulate tight junction protein expression and promote BSCB repair through the miR-501-5p/MLCK signaling pathway.

Although there is some understanding of how Exos promote angiogenesis and BSCB repair, further investigation is necessary to elucidate their mechanisms for better therapeutic efficacy.

### 3.5 Regulate the extracellular matrix (ECM)

The ECM is integral to the reparative processes following SCI (112). The lack of nerve regeneration is largely due to the absence of intrinsic nerve growth programs and the development of glial scars (113, 114). Exos, as essential mediators of intercellular communication, possess significant potential in facilitating ECM remodeling post-SCI (115, 116).

Exos play a crucial role in maintaining spinal stability by modulating the synthesis and degradation of collagen, regulating fibronectin levels, and influencing other components of the ECM. This modulation leads to alterations in both the structure and function of the ECM, while simultaneously inhibiting the release of inflammatory mediators and reducing the formation of glial scars. As a result, a conducive microenvironment for nerve regeneration is established (117, 118). Additionally, Exos can transport matrix metalloproteinases (MMPs) or their inhibitors, such as tissue inhibitors of metalloproteinases (TIMPs) (119), thereby managing ECM degradation and reconstruction by regulating the balance of these molecules (120). During tissue repair and regeneration, Exos enhance pathological microenvironments by preventing or mitigating scar tissue formation, thereby promoting repair (116). Liu et al. (121) demonstrated through animal studies that bone marrow mesenchymal stem cell-derived Exosomes (BMSC-Exos) effectively suppress inflammation following traumatic SCI, inhibit the activation of A1 neurotoxic reactive astrocytes, reduce glial scar formation, and facilitate nerve regeneration. In another animal experiment, Cheng et al. also found that human umbilical cord mesenchymal stem cells (HucMSC-EX) exosomes-embedded gelatin foam delivered miRNAs or proteins to inhibit the expression of chondroitin sulfate proteoglycan (CSPG) synthesis-related genes, while upregulating the activity of metalloproteinases such as ADAMTS to promote their degradation. On the other hand, it directly blocked the activation of CSPG receptors PTPσ and NgR, inhibited the downstream RhoA/ROCK pathway, and released the inhibition of axon growth. Gelatin sponge scaffolds can enhance the regulatory effect by sustained-release Exos and guide their directional distribution, and ultimately improve the microenvironment of nerve regeneration (122). Singh et al. found that Rab27a could mediate the release of CSPG-containing EVs from astrocytes, increase CSPG expression through the Rho/ROCK pathway, affect pAkt and β-tubulin III levels, and promote axonal degeneration and glial scar formation. This suggests that Rab27a-related mechanisms in Exos affect the content and distribution of CSPG in the ECM, which in consequence affects the repair process after SCI. Inhibition of Rab27a-mediated EVs release may reduce CSPG deposition, inhibit glial scar formation, and create a better ECM environment for nerve regeneration (123). Another team found that Exos secreted by UTX-depleted vascular endothelial cells carried miR-467b-3p, which transferred to macrophages, inhibited PTEN expression, activated PI3K/AKT/mTOR signaling pathway, and promoted macrophage polarization to anti-inflammatory M2 type, reducing inflammatory response. It can reduce the destruction of ECM by inflammation, and at the same time may promote the repair and remodeling of ECM, providing a more favorable microenvironment for nerve regeneration (100). In addition, some Exos can regulate the expression and function of BBB-related proteins in the blood. For example, arginine-glycine-aspartic acid (RGD) -modified Exos derived from CD163 + macrophages can deliver transforming growth factor β (TGF-β) to the neovascularization in the center of SCI, promote angiogenesis and stability of the blood-brain spinal barrier, and reduce the invasion of inflammatory cells and harmful substances. Maintaining the stability of ECM is beneficial to nerve regeneration (124).

Overall, Exos influence cellular behavior and tissue repair processes by modulating the composition, structure, and functionality of the ECM. These regulatory mechanisms are essential for the maintenance of normal tissue and recovery following injury.

## 4 Combination treatment strategy

**Figure 3:**
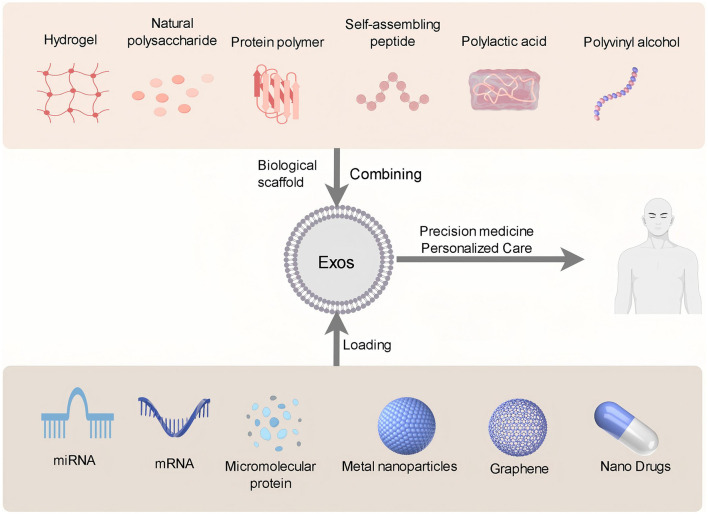
Exos combined with different materials can significantly improve the therapeutic effect on SCI. Combined with biological scaffolds, it can provide a stable release carrier for Exos and promote nerve tissue regeneration. Combined with nanomaterials, it can enhance targeted delivery and increase the concentration of Exos at the injury site. This combination method gives full play to the therapeutic potential of Exos to achieve precision medicine and personalized care.

The investigation into the integration of Exos with various materials for the treatment of SCI has attracted growing scholarly interest, owing to its potential to improve therapeutic outcomes and facilitate SCI repair. In this context, we outline several pivotal research directions and categories of materials (Table 3, Figure 3).

**Table 3:** Summary of nanodrug delivery systems.

**Types of nanodrug delivery systems**	**Type of loaded drugs**	**Therapeutic goal**	**Advantage**	**Disadvantage**
Exosome-liposome complex system (132, 179–181)	Small molecule anti-inflammatory drugs, nucleic acid drugs, neurotrophic peptides	Alzheimer's disease, Parkinson's disease, stroke	High drug loading, strong BBB penetration, and good compatibility	It is difficult to prepare, unstable and has a high mass production cost
Exosome-polymer nanoparticle composite system (132, 182–184)	Chemotherapy drugs, neurotrophic factors, targeted siRNA	Glioma, multiple sclerosis, SCI	Sustained-release, enhanced targeting, and good homology	It may cause inflammation, be easily cleared, and have a decreased targeting ability
Exosome-inorganic nanoparticle composite system (132, 185, 186)	Photothermal reagents, chemotherapy drugs, contrast agents	Glioma, brain metastases	It can be guided by imaging, has low toxicity and strong synergistic killing power	It is difficult to degrade, prone to accumulation, has a low encapsulation rate and is likely to clog blood vessels
Exosome-micellar composite system (132, 187, 188)	Hydrophobic chemotherapy drugs, fat-soluble antioxidants	Glioma, multiple sclerosis	Hydrophobic drugs have a high encapsulation rate and small particle size, making them easy to penetrate	Micelles are prone to disintegration, their structures are easily damaged, and there is a risk of transfer
Exosome-hydrogel composite system (132, 189, 190)	Neurotrophic factor, miRNA	Stroke, SCI	Sustainable release, supported by physical means, with low local toxicity	Micelles are prone to disintegration, their structures are easily damaged, and there is a risk of transfer
Exosome-metal-organic framework (MOF) composite system (132, 191, 192)	Chemotherapy drugs, immune siRNA, PET contrast agents	Glioma	High targeting, can respond to drug release, and also has imaging functions	The safety of the degradation products remains to be verified, their preparation is difficult, and the binding rate is low
Exosome-virus-like particle (VLPs) complex system (132, 193, 194)	Gene drugs, siRNA	Depression, neurodegenerative diseases	Strong uptake, high targeting, and capable of mucosal deliveryimaging functions	It may trigger an immune response, but the efficiency varies and the safety remains to be investigated

### 4.1 Combining Exos with biomaterials

Firstly, hydrogel, as a scaffold material, exhibits excellent biocompatibility and possesses loose and porous structural characteristics. It can serve as a carrier for Exos, thereby prolonging their residence time in specific areas and facilitating controlled release (106). Numerous studies have demonstrated that the combination of Exos with hydrogels promotes the survival and regeneration of nerve cells while reducing inflammatory responses (72, 73). Han et al. (125) utilized Exos combined with hydrogel to treat SCI, ensuring more reliable, convenient, and effective delivery of Exos to targeted regions. Guan et al. (126) combined M2-Exos with hydrogel, while Li et al. (127) combined MSC-Exos with hydrogel; both treatment approaches resulted in accelerated neuron and axon regeneration as well as significantly enhanced functional recovery in SCI rats. Secondly, bioscaffolds composed of natural polysaccharides, protein polymers, self-assembled peptides, and biocompatible polymers such as polylactic acid (PLA) and polyvinyl alcohol (PVA) have been extensively utilized in the repair of spinal cord injuries (128). Liu et al. (129) incorporated collagen scaffolds with Exos' surface to facilitate the retention of miR21-loaded Exos at the lesion site and ensure a sustained release of miR21 into cells. Zhang et al. (130) fused umbilical cord MSC-Exos with multifunctional collagen scaffolds to offer a versatile therapeutic approach for various diseases including SCI. The combination of Exos with these scaffold materials can augment the biological activity of the scaffold and promote nerve regeneration.

### 4.2 Combining Exos with nanomaterials

Exos is a natural nanocarrier secreted by a variety of cells. It has the characteristics of high stability, targeting, low immunogenicity, and good biocompatibility, and is suitable for various drug delivery and therapeutic applications (26, 28, 35, 131). Through genetic engineering and chemical modification techniques, drugs can be encapsulated inside or attached to the surface of Exos to construct targeted drug delivery systems (NDDS) that can specifically deliver drugs to certain types of cells or tissues (27, 132). The most common bioactive molecules loaded in Exos include miRNA, mRNA, proteins, and small molecules (39, 133). Moreover, engineered Exos can also be combined with metal nanoparticles, graphene, and other nanomaterials to enhance their targeting ability and bioavailability (134, 135). In the treatment of nervous system diseases specifically, Exos have emerged as promising carriers for delivering drugs due to their inherent capability to cross the BBB and BSCB (111, 136). Guo et al. (137) delivered Exos loaded with phosphatase and tenin homologous siRNA to SCI rats and found that Exos could cross the BBB and migrate to the injured spinal cord area, improving motor function, sensory function, and faster recovery of urinary reflex. Cui et al. (138) on the other hand, utilized immune Exos loaded nano micelles capable of crossing the BBB for treating glioblastoma, which not only exhibited improved efficacy but also prevented postoperative recurrence. In another study conducted by Gao et al. (139) M2-Exos loaded with berberine were employed for treating mice with SCI, resulting in significant improvement in motor function. As carriers for NDDS, Exos exhibit great potential and application prospects. With further research advancements, Exos-based therapies will find wider utility in precision medicine, personalized therapy, and other related fields.

## 5 Current status of clinical research

Clinical studies investigating the therapeutic potential of Exos in SCI are currently underway, and although still in its early stages, significant progress has been made. Several small-scale clinical trials have been conducted to assess the safety and initial efficacy of Exos (140). Most studies have demonstrated that short-term treatment with Exos is well-tolerated (21, 141), with no reports of serious adverse reactions (142, 143). These trials typically involve the utilization of MSC-derived Exos to evaluate their application in patients with SCI (144, 145).

Akhlaghpasand et al. conducted the initial phase I clinical trial of Exos in treating SCI, wherein intradermal injection of allogenic Exos derived from human umbilical cord MSC was administered to patients with acute SCI. The findings demonstrated favorable tolerability and the absence of significant adverse reactions associated with Exos (Iranian Registry of Clinical Trials, IRCT20200502047277N1) (146). This pioneering study not only establishes the safety profile of stem cell Exosome therapy for SCI in human subjects but also highlights its potential clinical benefits, instilling renewed hope among SCI patients and providing a crucial scientific foundation for the medical community.

Overall, Exos exhibits promising safety and efficacy in SCI treatment; however, further evidence is required to ascertain its clinical translational potential (147).

## 6 The challenges and prospects of Exos therapy for SCI

### 6.1 The challenges faced

In the progress of researching Exos-based SCI therapies, despite significant achievements, the field still faces numerous challenges and unanswered questions. Firstly, a major issue lies in the source and quality control of Exos (32, 131). Exos derived from different cell origins may exhibit substantial variations in composition, function, and therapeutic effects (148). Currently, various Exos separation technologies have been developed based on size, density, compatibility, and surface protein characteristics (149), but large-scale mass production (13) and ensuring the purity, stability, and biological activity of Exos remain crucial issues that need to be addressed. Additionally, during storage and transportation processes, Exos are prone to aggregation and degradation, which can impact their therapeutic efficacy (150, 151). Secondly, the therapeutic mechanism of Exos is not fully understood. While it is known that Exos can exert therapeutic effects by delivering bioactive molecules, little is known about their specific signaling pathways or cell-cell interactions among other mechanisms (16, 115). This lack of understanding makes it challenging to optimize and personalize Exos therapy. Furthermore, targeted delivery of Exos also poses a challenge. Despite improvements in targeting ability through combining with biological scaffolds, the presence of BSCB at the SCI site, along with the complex microenvironment, still presents significant obstacles for efficient targeted delivery (152). Lastly, long-term efficacy and safety concerns regarding Exos therapy cannot be ignored. Although short-term animal experiments have shown certain effectiveness of Exos treatment (122, 153), further investigation is required to assess long-term effects and potential complications such as tumor formation and neurodegeneration (34, 154). Designing well-designed clinical trials to evaluate the safety and effectiveness of Exos therapy is an important step toward the clinical translation of the therapy.

Future research should focus more on these issues, pushing the field forward through technological innovation and rigorous scientific validation, ultimately providing more effective treatment options for SCI patients.

### 6.2 Prospects

With the ongoing advancement of Exos research, significant breakthroughs are expected in the treatment of SCI. The future research directions mainly focus on optimizing the methods for the separation and purification of Exos to enhance both yield and quality. Furthermore, investigating the underlying mechanisms of Exos will provide a solid theoretical foundation for their clinical application. Additionally, developing targeted therapeutic strategies for Exos and improving their efficacy aims to boost treatment outcomes. Lastly, carrying out large-scale clinical trials is essential to validate the safety and effectiveness of Exos in treating SCI.

## 7 Conclusion

As a severe medical condition, SCI not only imposes significant physical and psychological burdens on patients, but also places immense pressure on the social healthcare system. In recent years, there has been considerable attention given to Exos-based SCI therapies due to their distinctive biological characteristics and therapeutic potential. Through an in-depth analysis of relevant literature, it can be observed that utilizing Exos as carriers for specific miRNAs exhibits unprecedented therapeutic promise. Firstly, the advantage of Exos as bioactive carriers lies in their efficient ability to transport various biomolecules such as proteins, mRNA, and miRNA between cells. Particularly when these Exos are enriched with specific classes of miRNAs, they demonstrate remarkable effects in regulating nerve regeneration, inhibiting inflammatory responses, and promoting angiogenesis. Furthermore, research has revealed hypoxia preconditioning as a potential method to enhance the efficacy of Exos therapy. This finding provides a crucial experimental basis for improving the efficiency of Exos treatment for SCI. However, despite the significant potential shown by Exos-based therapies, their clinical application still faces numerous challenges. In conclusion, although Exos-based SCI treatment is still at an early stage of research development, it has demonstrated substantial therapeutic potential and promising prospects for further advancement. Future research should focus on overcoming existing technical barriers and expediting the transition from this emerging treatment to clinical application. Additionally, continuous exploration and optimization of Exos conte ts, nparticularly in the search for more efficient combinations with miRNA and nanomaterials, will further propel advancements in this field toward providing substantial assistance to patients with SCI.
